# Immune microenvironment features and efficacy of PD‐1/PD‐L1 blockade in non‐small cell lung cancer patients with *EGFR* or *HER2* exon 20 insertions

**DOI:** 10.1111/1759-7714.13748

**Published:** 2020-11-18

**Authors:** Kaiyan Chen, Guoqiang Pan, Guoping Cheng, Fanrong Zhang, Yanjun Xu, Zhiyu Huang, Yun Fan

**Affiliations:** ^1^ The Cancer Hospital of the University of Chinese Academy of Sciences (Zhejiang Cancer Hospital) Hangzhou China; ^2^ Institute of Basic Medicine and Cancer (IBMC) Chinese Academy of Sciences Hangzhou China; ^3^ Department of Thoracic Medical Oncology Zhejiang Cancer Hospital Hangzhou China; ^4^ Department of Pathology Zhejiang Cancer Hospital Hangzhou China; ^5^ Department of Breast Surgery Zhejiang Cancer Hospital Hangzhou China; ^6^ Department of Oncology The First Clinical Medical College of Wenzhou Medical University Wenzhou China

**Keywords:** *EGFR*, *HER2*, immunotherapy, NSCLC, PD‐L1

## Abstract

**Background:**

Insertions in exon 20 (Ex20ins) of epidermal growth factor receptor (*EGFR*) and human epidermal growth factor receptor 2 (*HER2)* are relatively insensitive to first‐ and second‐generation EGFR‐tyrosine kinase inhibitors (TKIs) in non‐small cell lung cancer (NSCLC). This study aimed to investigate the immune microenvironment features and efficacy of PD‐1/PD‐L1 blockade of NSCLC with *EGFR* and *HER2* Ex20ins.

**Methods:**

Clinical characteristics, coexisting mutations, and outcomes to EGFR‐TKIs and immune checkpoint blockade were reviewed for NSCLC patients with exon 20 mutations of *EGFR* or *HER2*. Data obtained included the molecular spectrum (extended genotyping for mutations in 324 cancer‐related genes), as well as tumor mutational burden (TMB), PD‐L1 protein expression, and the abundance of CD4+ and CD8+ tumor‐infiltrating lymphocytes (TILs).

**Results:**

A total of 1270 NSCLC patients were identified. Of these, 504 (39.7%) cases had *EGFR* mutations and 6.9% (35/504) of them had *EGFR* Ex20ins. Meanwhile, 21 (1.7%) cases with *HER2* Ex20ins were detected. Comprehensive genomic profiling identified A767_V769dup variant (25.0%) was the most common type in tumors with *EGFR* Ex20ins. Co‐occurring mutations were not uncommon including *TP53* (45%), *PIK3CA* (20%), *CDKN2A* (10%), and *EGFR* amplification (20%). The average TMB was 3.3 mutations/megabase. PD‐L1 expression in patients with *EGFR* Ex20ins was significantly higher than for those with *HER2* mutations (48.6% vs. 19.0%, *P* = 0.027). High TMB and PD‐L1 expression was independently associated with significantly poor prognosis (*P* = 0.025, *P* = 0.045, respectively) while there was no association between CD4+/CD8+ TILs and prognosis in *EGFR* or *HER2* mutant NSCLC. Finally, patients harboring *EGFR* Ex20ins seemed to be sensitive to PD‐1/PD‐L1 blockage whereas it showed limited efficacy in patients with *HER2* Ex20ins.

**Conclusions:**

NSCLC patients with *EGFR*/*HER2* Ex20ins had similar genomic characteristics and distinct immune features. Patients with *EGFR* Ex20ins had significantly higher PD‐L1 expression than those with *HER2* mutations, which may be the potential reason for the different responses to PD‐1/PD‐L1 blockage.

## Graphical abstract

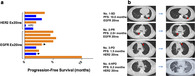

NSCLC patients with EGFR/HER2 Ex20ins had similar genomic characteristics and distinct immune features when compared to common *EGFR* mutations. Patients with EGFR Ex20ins had significantly higher PD‐L1 expression than those with HER2 mutations, which may be the potential reason for the different responses to PD‐1/PD‐L1 blockage.

## Introduction

Lung cancer is the most common cause of cancer‐related death worldwide,^1^ with non‐small cell lung cancer (NSCLC) accounting for 75% of all lung cancer cases.^2^ Most patients with NSCLC harboring sensitizing epidermal growth factor receptor *(EGFR)* mutations confer a high response rate of 70%–80% to EGFR‐tyrosine kinase inhibitors (TKIs) such as erlotinib, gefitinib, and afatinib.^3^, ^4^, ^5^ However, approximately 4%–12% of *EGFR*‐mutant NSCLC tumors have in‐frame insertions within exon 20, named *EGFR* Ex20ins, and are generally insensitive to first‐ or second‐generation EGFR‐TKIs due to the modified structures of their kinase domains (except for A763_764insFQEA),^6^, ^7^, ^8^ resulting in a dismal prognosis.^9^, ^10^

Currently, a family of Ex20ins has been described in the human epidermal growth factor receptor 2 gene (*HER2*, also known as *ERBB2*), which consists of 90% of *HER2* alterations in NSCLC, and approximately 2%–4% of patients with NSCLC harbor these mutations.^11^, ^12^ Exon 20 alterations of *EGFR* and *HER2* have similar crystal structures and biological functions, including the α‐C helix (residues 762–766 in *EGFR* and 770–774 in *HER2*) and the loop following the α‐C helix (residues 767–774 in *EGFR* and 775–783 in *HER2*).^12^, ^13^ Generally, mutations in exon 20 have shown constitutive phosphorylation of kinase domain, resulting in downstream activation of the PI3K‐AKT and MAPK pathways, which induce tumor initiation and development.^12^ Similar to the patients with *EGFR* Ex20ins, treatment with the EGFR/HER2‐selective TKIs (eg, afatinib or dacomitinib) have reported limited success in patients with exon 20 mutations of *HER2*.^12^ However, several next‐generation EGFR‐TKIs (some with pan‐HER activity) have demonstrated preclinical activity against mutations in exon 20 of either *EGFR* or *HER2* and are in clinical development (TAK‐788, osimertinib, poziotinib).^12^, ^14^ To date, no EGFR‐TKIs are approved for these patients and their diversity of structures suggests that different insertion events may have divergent responsiveness to various TKIs.^13^ Thus, identifying the clinical features and new treatment response is of paramount importance.

Immune checkpoint inhibitors (ICIs) such as programmed cell death‐1 (PD‐1)/programmed cell death ligand‐1 (PD‐L1) antibodies have led to unprecedented durable clinical benefit for NSCLC, but response rates are low for patients with targetable driver mutations.^15^ Biomarker studies have revealed a significant correlation between PD‐L1 expression and the likelihood of a response to PD‐1 inhibitors, whereas *EGFR* mutation appears to be a negative predictive factor.^16^, ^17^ Given that most sensitizing *EGFR* mutations are not responsive to PD‐1 blockade either exon 19 deletions or L858R in exon 21; however, it is unclear whether such treatment is also without benefit in patients with uncommon *EGFR* mutations.^18^ Currently, few reports with integrated analyses interpret the underlying mechanism of the response to PD‐1/PD‐L1 inhibitors in the Ex20ins subgroup. Therefore, there is a substantial clinical need to identify new therapies to overcome the innate drug resistance of NSCLC tumors harboring exon 20 insertions in *EGFR* or *HER2*.

The present study aimed to evaluate the genomic and immune characteristics of NSCLC patients with *EGFR* or *HER2* exon 20 mutations from a large dataset with molecular spectrum, tumor mutational burden (TMB), PD‐L1 expression, the CD4+ and CD8+ tumor‐infiltrating lymphocytes (TILs) infiltration, as well as the efficacy of immune checkpoint inhibitor and TKIs.

## Methods

### Patients

Among 1270 NSCLC patients, 35 patients carrying *EGFR* Ex20ins and 21 patients harboring *HER2* Ex20ins in Zhejiang Cancer Hospital between April 2016 and September 2018 were retrospectively enrolled. Clinical data were obtained from the electronic medical record database. Tumor response was examined by computed tomography (CT) and evaluated according to the Response Evaluation Criteria in Solid Tumors (RECIST) version 1.1. The study protocol was approved by the ethics committee of Zhejiang Cancer Hospital.

### Immunohistochemistry

Immunohistochemistry (IHC) analyses were carried out according to standard protocols. Immunohistochemical staining for PD‐L1 was performed using Dako PD‐L1 IHC 22C3 pharmDx kit and a Dako Autostainer Link 48 with standard antigen retrieval methods. The antibodies used for TILs infiltration evaluation were antihuman CD4 (clone: B468A1, diluted at 1:200, Santa Cruz, Texas, USA) and antihuman CD8 (clone 144B, diluted at 1:100, Abcam, Cambridge, UK). All IHC stained sections were reviewed by two independent pathologists who were blinded to the clinical information.

Tumors with ≥1% of tumor cells stained in membrane were considered positive for PD‐L1.^7^ PD‐L1 immunohistochemistry (Clone 22C3) was graded by a tumor positive score (TPS) system. Consistent with a previous study,^19^ our study examined CD4 and CD8 staining on lymphocytes as the proportion of positive cells among all nucleated cells, and soring was recorded as negative (<10%) or positive (≥10%).

### Genomic analysis

Collected formalin‐fixed, paraffin‐embedded tumor specimens underwent histological review, and only those containing sufficient tumor cells as shown by hematoxylin‐eosin staining were subjected to nucleic acid extraction. A total of 31 patients including 20 cases with *EGFR* mutation and 11 cases with *HER2* mutation received comprehensive genomic profiling (CGP) testing to determine the genomic status, which targets 324 cancer‐associated genes. Patient samples were evaluated for genomic alterations, including base pair substitutions, insertions/deletions (indels), copy number alterations, and rearrangements. TMB was characterized in 31 individuals as the number of somatic base substitution or indel alterations per megabase (Mb) per previously described methods.^20^ In line with a previous study,^8^ the cutoff value of TMB was five mutations/Mb in patients with Ex20ins. In addition, the remaining 15 patients with *EGFR* Ex20ins and 10 with *HER2* mutation were tested for cancer‐associated genes (*EGFR/ALK/ROS1/KRAS/NRAS/BRAF/RET/HER2/PIK3CA/MET*) by polymerase chain reaction. Tumor specimens and genomic DNA were isolated using the AMRS DNA Sample Preparation Kit (Amoy Diagnostics, Xiamen, People's Republic of China) following the manufacturer's instructions.

### Statistical analysis

Statistical analyses were performed using SPSS version 20.0 (Chicago, IL) and GraphPad Prism (version 7.01). The chi‐square test or Fisher's exact test were used to assess the significance among categorical variables. The survival curves were generated by Kaplan‐Meier estimator and the Cox proportional hazards regression model was adopted to determine the hazard ratio (HR). Variables of univariate analysis with *P* < 0.1 were included in the multivariate Cox regression analysis. Statistical significance was considered as *P* ≤ 0.05 using a two‐sided test.

## Results

### Clinical characteristics

Among 1270 NSCLC patients, 504 (39.7%) cases had *EGFR* mutations. A total of 35 cases with *EGFR* Ex20ins and 21 cases harboring *HER2* Ex20ins were identified. Of the patients with *EGFR*/*HER2* Ex20ins, the median age was 56 years old (range: 29–80 years); 57.1% (32 patients) were female, 71.4% (40 patients) were younger than 65 years, 32.1% (18 patients) were current or former smokers, 85.7% (48 patients) were diagnosed with stage IV, 46.4% (26 patients) had family history, 50.0% (28 patients) previously received TKIs, 89.3% (50 patients) previously received chemotherapy, 53.6% (30 patients) were given third‐line or beyond therapy, and 91.1% (51 patients) had a performance status (PS) score of 0 to 1. No differences on above baseline characteristics were found between *EGFR* and *HER2* mutant NSCLC (*P* > 0.05, Table 1). Clinical demographics of this cohort are summarized in Table 1.

**Table 1:** The clinicopathological factors in NSCLC patients with *EGFR* or *HER2* Ex20ins

Parameters	All cases (*N* = 56)	*EGFR* Ex20ins	*HER2* EX20ins	*P*‐value
Gender				
Male	24 (42.9%)	15 (42.9%)	9 (42.9%)	1.000
Female	32 (57.1%)	20 (57.1%)	12 (57.1%)	
Age, year				
<65	40 (71.4%)	22 (62.9%)	18 (85.7%)	0.067
≥65	16 (28.6%)	13 (37.1%)	3 (14.3%)	
Smoking status				
Never	38 (67.9%)	25 (71.4%)	13 (61.9%)	0.460
Ever/current	18 (32.1%)	10 (28.6%)	8 (38.1%)	
Performance status				
0–1	51 (91.1%)	31 (88.6%)	20 (95.2%)	0.717
2	5 (8.9%)	4 (11.4%)	1 (4.8%)	
Family history				
Yes	26 (46.4%)	13 (37.1%)	13 (61.9%)	0.072
No	30 (53.6%)	22 (62.9%)	8 (38.1%)	
Disease stage				
IIIb	8 (14.3%)	4 (11.4%)	4 (19.0%)	0.430
IV	48 (85.7%)	31 (88.6%)	17 (81.0%)	
PD‐L1 status				
<1%	36 (64.3%)	18 (51.4%)	17 (81.0%)	***0.027***
≥1%	20 (35.7%)	17 (48.6%)	4 (19.0%)	
CD4 expression				
Negative	34 (60.7%)	22 (62.9%)	12 (57.1%)	0.672
Positive	22 (39.3%)	13 (37.1%)	9 (42.9%)	
CD8 expression				
Negative	22 (39.3%)	14 (40.0%)	8 (38.1%)	0.888
Positive	34 (60.7%)	21 (60.0%)	13 (61.9%)	
Lines of treatment				
First/second	26 (46.4%)	15 (42.9%)	10 (47.6%)	0.729
Third/posterior line	30 (53.6%)	20 (57.1%)	11 (52.4%)	
Previous TKI therapy				
Yes	28 (50.0%)	15 (42.9%)	13 (61.9%)	0.168
No	28 (50.0%)	20 (57.1%)	8 (38.1%)	
Previous chemotherapy therapy				
Yes	50 (89.3%)	30 (85.7%)	20 (95.2%)	0.265
No	6 (10.7%)	5 (14.3%)	1 (4.8%)	
Total	56	35 (62.5%)	21 (37.5%)	

TKI, tyrosine kinase inhibitors.

### Genomic characteristics

**Figure 1:**
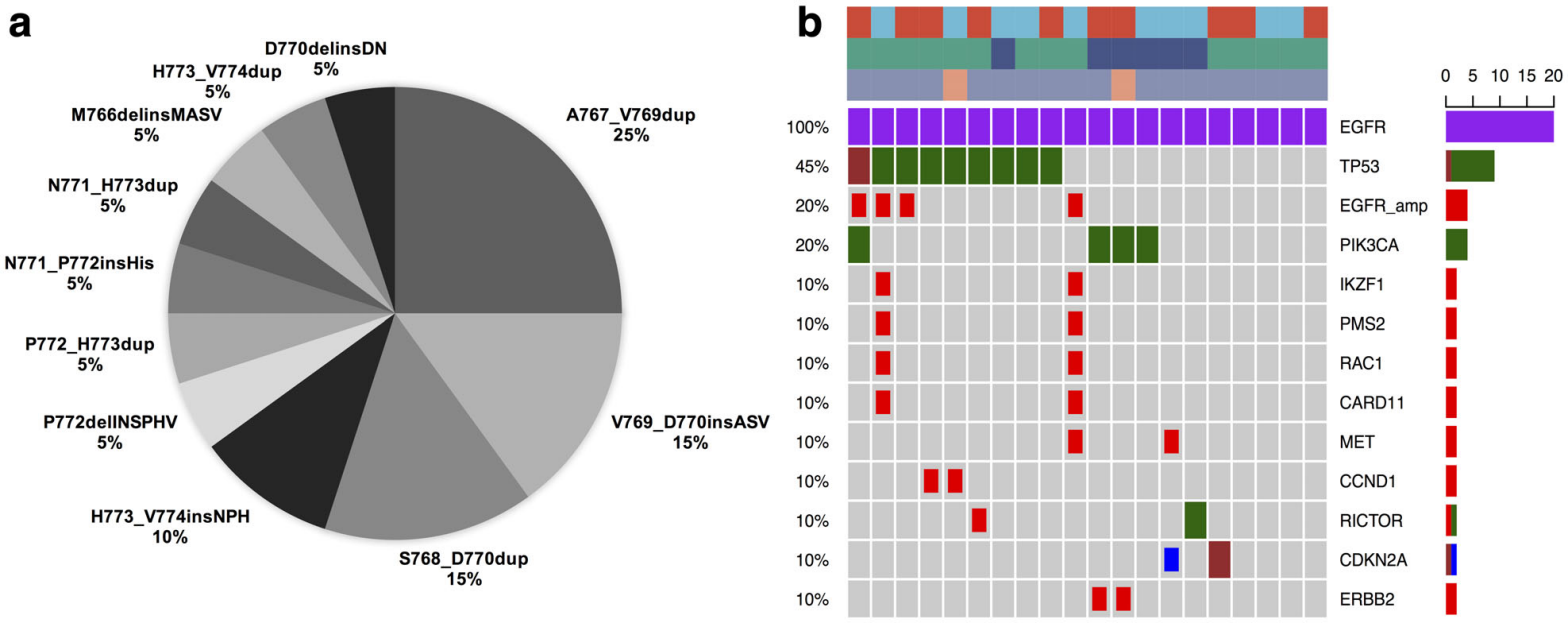
(**a**) The subtype of *EGFR* Ex20ins variants among NSCLC. (**b**) The distribution of *EGFR* Ex20ins in NSCLC patients with concurrent mutations. Somatic gene mutations detected with comprehensive genomic profiling testing covering 324 genes in lung cancer specimens positive for exon 20 mutation of *EGFR*. Each column corresponds to one of the 20 patients. Alterations: 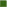, Missense; 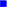, CN_del; 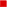, CN_amp; 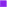, Indel; 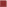, Splice_site. Gender: 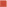, Male; 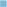, Female. Age: 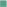, <65; 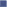, >=65. Stage: 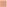, III; 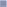, IV.

CGP identified A767_V769dup variant was the most frequent, comprising 25.0% of *EGFR* Ex20ins cases. In addition, *EGFR* Ex20ins patients had co‐occurring mutations including *TP53* (45%), *PIK3CA* (20%), *CDKN2A* (10%), and *EGFR* amplification (20%). Figure 1a shows the subtype of *EGFR* Ex20ins variants among NSCLC and Fig 1b indicates the distribution of *EGFR* Ex20ins in patients with concurrent mutations. Notably, the average TMB of *EGFR/HER2* Ex20ins was 3.3 mutations/Mb；and eight cases (25.8%) had TMB more than five mutations/Mb. Further multivariate analysis suggested that TMB was an independent prognostic factor after adjusting for clinicopathological factors (HR = 4.95, 95% confidence interval [CI]: 1.46–6.84, *P* = 0.025, Table 2). With regard to *HER2* Ex20ins mutants, A775_G776insYVMA variant was the most common type, making up 80.9% of *HER2*‐mutant cases. The most common concurrent mutations were *TP53* and *PIK3CA*.

**Table 2:** Univariate and multivariate Cox regression analyses of prognostic factors for survival in NSCLC patients with *EGFR* or *HER2* Ex20ins

	Univariate analysis		Multivariate analysis	
Parameters	HR (95% CI)	*P‐*value	HR (95% CI)	*P*‐value
Gender				
Male	1.00		1.00	
Female	0.38 (0.13–1.09)	0.073	0.22 (0.11–1.43)	0.052
Age, year				
<65	1.00			
≥65	0.80 (0.26–2.45)	0.702		
Smoking status				
Never	1.00			
Ever/current	1.73 (0.65–4.62)	0.276		
Performance status				
0–1	1.00			
2	0.89 (0.31–2.59)	0.835		
Family history				
Yes	1.00			
No	0.51 (0.18–1.39)	0.188		
Disease stage				
IIIb	1.00			
IV	2.92 (0.27–3.99)	0.350		
PD‐L1 expression				
<1%	1.00		1.00	
≥1%	4.67 (1.80–12.1)	***0.002***	6.21 (1.05–8.22)	***0.045***
CD4 expression				
No	1.00			
Yes	0.70 (0.25–1.98)	0.506		
CD8 expression				
No	1.00			
Yes	0.46 (0.18–1.17)	0.104		
TMB				
Low (<5 mutation/Mb)	1.00		1.00	
High (≥5 mutation/Mb)	6.7 (1.59–8.41)	***0.019***	4.95 (1.46–6.84)	***0.025***

CI, confidence interval; HR, hazard ratio; Mb, megabase; TMB, tumor mutational burden.

### Immune microenvironment feature

**Figure 2:**
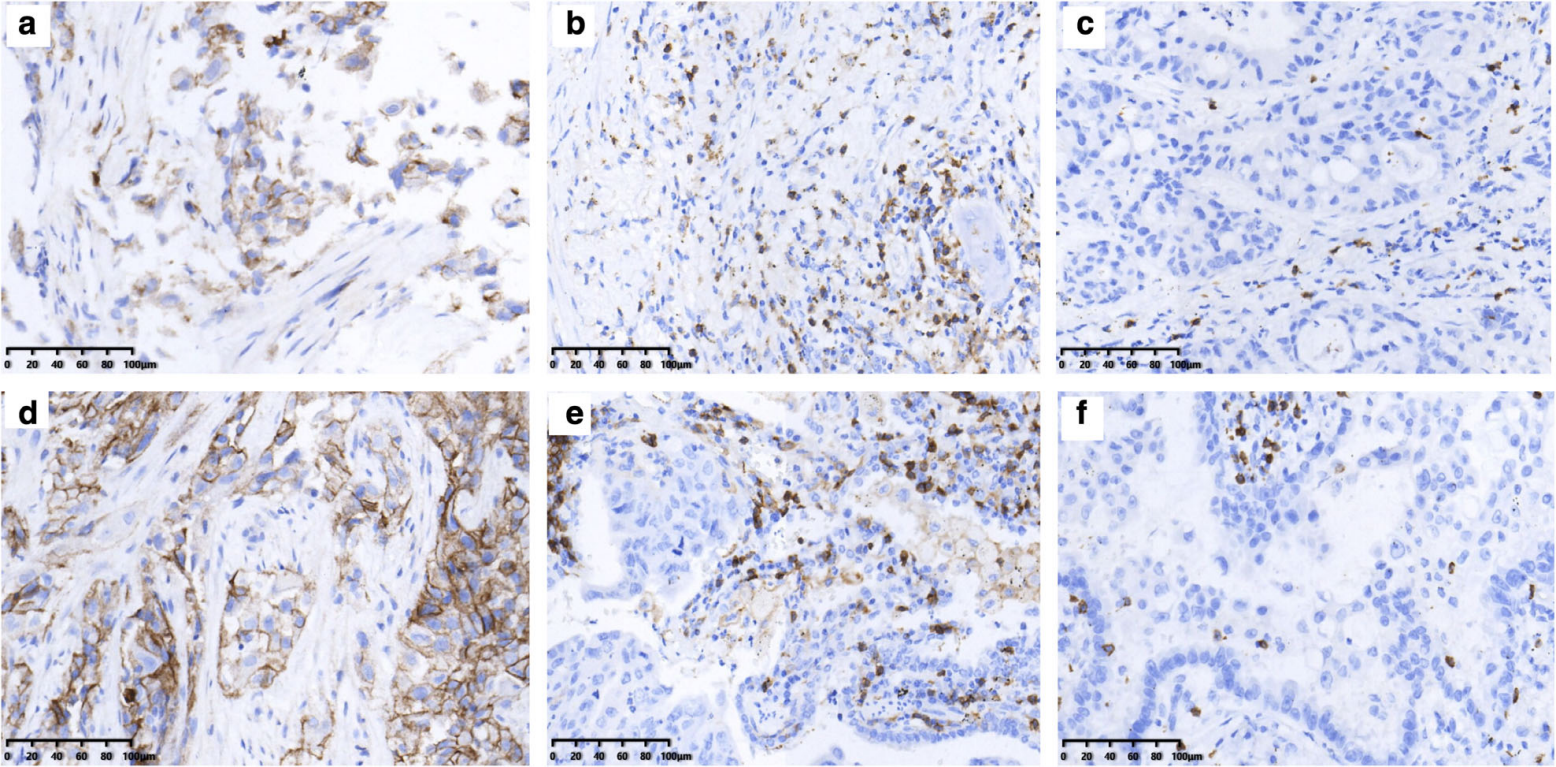
The expression of PD‐L1 and infiltration of CD4+, CD8+ T cells within NSCLC patients harboring Ex20ins of *EGFR* or *HER2*. NSCLC tumors with *EGFR* Ex20ins: (**a**) Positive expression of PD‐L1; (**b**) presence of CD4+ TILs; (**c**) presence of CD8+ TILs. NSCLC tumors with *HER2* Ex20ins: (**d**) Positive expression of PD‐L1; (**e**) presence of CD4+ TILs; and (**f**) presence of CD8+ TILs. (*200).

**Figure 3:**
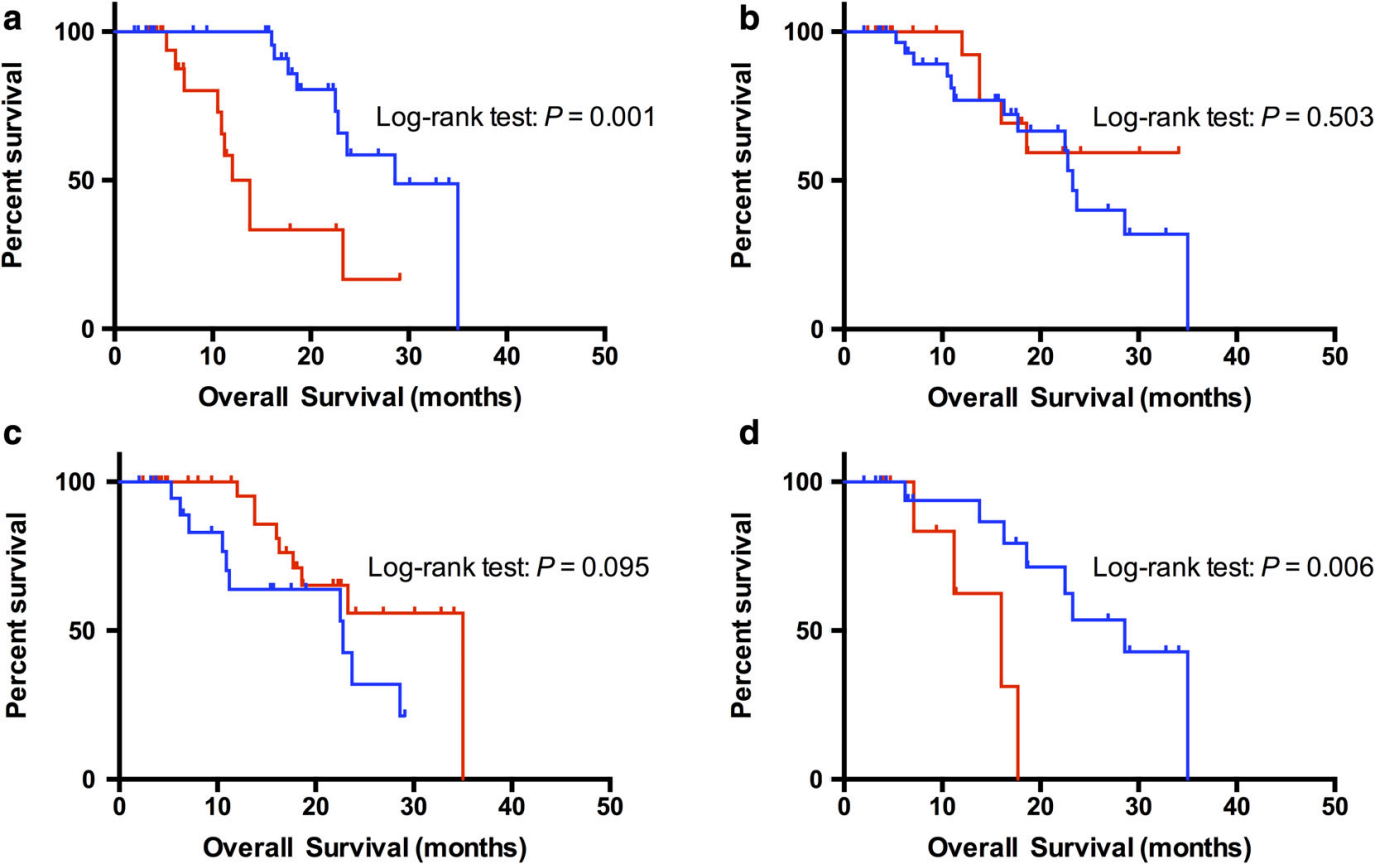
Kaplan‐Meier curves of the median overall survival (OS) of Ex20ins NSCLC patients stratified by PD‐L1 expression, TIL infiltration and TMB data. (**a**) NSCLC patients with PD‐L1 expression had a median OS time of 12.0 months, which was shorter than the median OS time of patients with negative expression (12.0 vs. 28.6 months, *P* = 0.001) 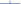, PD‐L1 Negative; 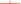, PD‐L1 Positive. (**b**,**c**) Neither the infiltration of CD4+ TILs nor that of CD8+ TILs showed prognostic value in *EGFR* or *HER2* mutant NSCLC (*P* = 0.503, *P* = 0.095, respectively) 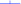, CD4 Negative; 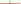, CD4 Positive; 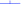, CD8 Negative; 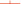, CD8 Positive. (**d**) NSCLC patients with TMB‐high exhibited a median OS time of 16.0 months, which was shorter than those in TMB‐low group (16.0 vs. 28.6 months, *P* = 0.006) 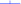, TMB‐low; 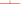, TMB‐high.

PD‐L1 staining as well as the presence and absence of CD4+ and CD8 + TILs were used to show the tumor immune microenvironment feature (Fig 2). In total, 48.6% (17/35) of the NSCLC patients showed positive PD‐L1 expression in TCs of *EGFR* Ex20ins tumors; this percentage was markedly higher than that in *HER2*‐mutant patients (48.6% vs. 19.0%, *P* = 0.027), whereas the distribution of CD4+ or CD8+ TILs was similar in these two groups (*P* = 0.672, *P* = 0.888, Table 1). Among the patients with *EGFR* and *HER2* Ex20ins, the median OS was significantly shorter in the PD‐L1‐positive group than in the PD‐L1‐negative group (12.0 vs. 28.6 months, *P* = 0.001, Fig 3) and PD‐L1 was identified as an independent predictor (HR = 6.21, 95% CI: 1.05–8.22, *P* = 0.045, Table 2). Neither the infiltration of CD4+ TILs nor that of CD8+ TILs showed prognostic value in *EGFR* and *HER2* mutant NSCLC (*P* = 0.503, *P* = 0.095, respectively; Fig 3). Moreover, no relationship between PD‐L1 expression and TILs infiltration was found in patients with Ex20ins (*P* = 0.888, *P* = 0.672, respectively).

### Treatment and survival outcomes

**Figure 4:**
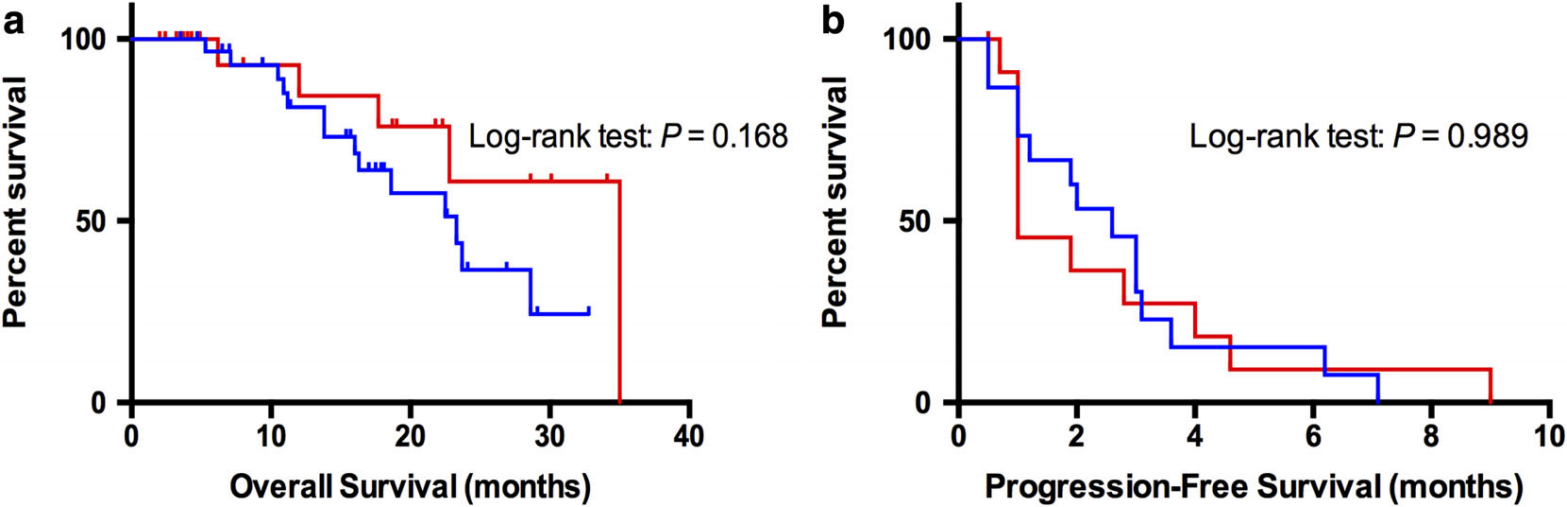
Kaplan‐Meier curves of the median OS among NSCLC patients with Ex20ins (**a**) 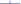, EGFR 20ins; 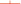, HER2 20ins, and progression‐free survival (PFS) of Ex20ins NSCLC patients to TKIs treatment (**b**) 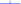, EGFR 20ins; 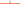, HER2 20ins. There was no survival difference between *EGFR* exon 20 insertion and *HER‐2* exon 20 insertion (23.3 vs. 35.0 months, *P* = 0.168). Also, PFS was similar between population with *EGFR* and *HER2* Ex20ins who received TKI therapy (2.6 vs. 1.0 months, *P* = 0.989).

Median OS for the entire population with *EGFR* or *HER2* exon 20 mutations was 23.3 months (95% CI: 18.1–28.2). There was no survival difference between *EGFR* exon 20 insertion and *HER‐2* exon 20 insertion (23.3 vs. 35.0 months, *P* = 0.168, Fig 4a), and half of the patients had previously received EGFR‐TKIs. Specifically, 15 patients (42.9%) with tumors expressing an Ex20ins of *EGFR* were treated with targeted therapeutic strategies (eg, icotinib, gefitinib or afatinib) with an objective response rate (ORR) of 13.3%, and 61.9% of *HER2*‐mutant cases received pyrotinib or afatinib with an ORR of 11.1%. Progression‐free survival (PFS) was similar between both groups (2.6 vs. 1.0 months; *P* = 0.989, Fig 4b). Although the efficacy of TKIs was dismal, one patient with *HER2* Ex20ins had a partial response (PR) to pyrotinib with a PFS of 9.0 months. Additionally, most patients (*N* = 40) received carboplatin or cisplatin/pemetrexed with an ORR of 30.0%, and were then treated with docetaxel with an ORR of 10.0% when their disease progressed. The efficacy of chemotherapy was similar between NSCLC patients with Ex20ins and wild‐type.^3^, ^4^

**Figure 5:**
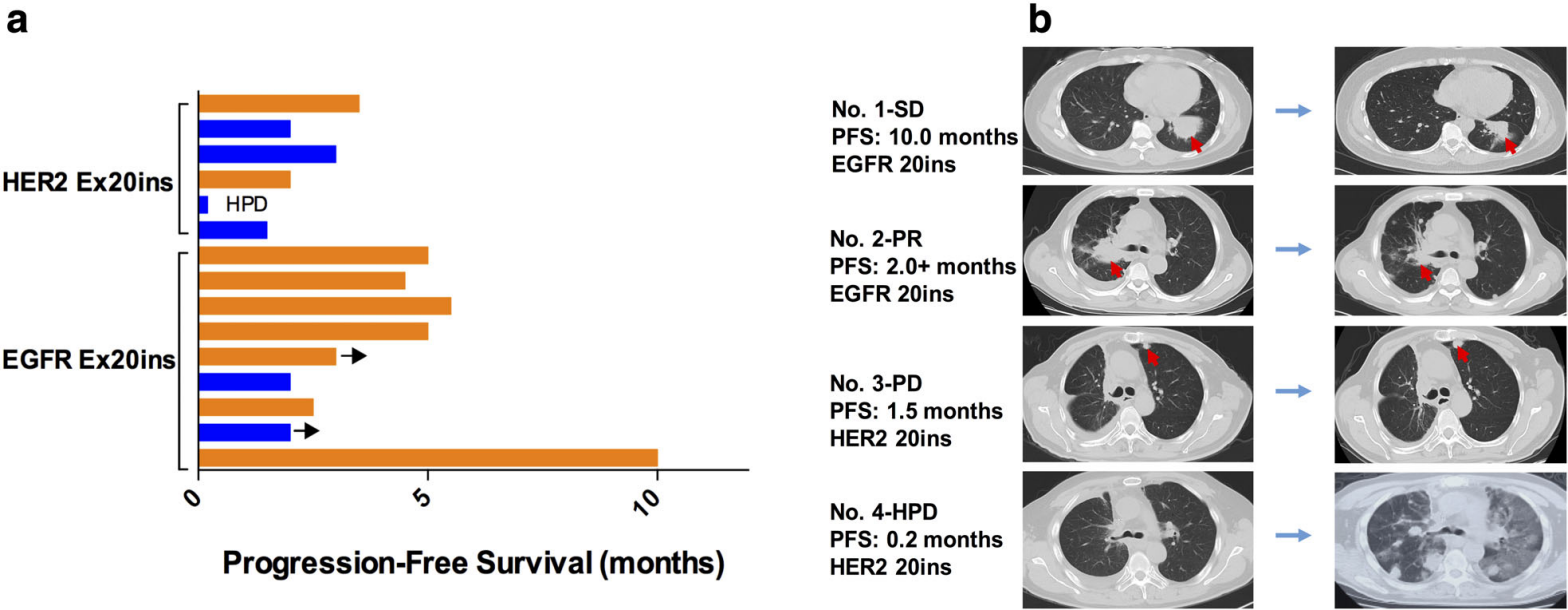
(**a**) Swimmer plot for duration of disease stability or response to immune checkpoint inhibitors in patients with exon 20 mutation of *EGFR* or *HER2*. A total of 15 patients received immunotherapy including nine patients with *EGFR* Ex20ins and six with *HER2* Ex20ins. Bar length indicates the duration of immunotherapy treatment for each patient, with the response observed before treatment failure indicated on the right. The origin corresponds to treatment start date, and the arrow indicates an ongoing response at the time of data censoring 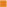, PD‐L1 ≥1%; 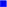, PD‐L1 <1%. (**b**) Computed tomography scans of the thorax performed prior (baseline) and after anti‐PD‐1 treatment onset in four patients. The red arrow shows the lung lesion.

To evaluate the clinical response to PD‐1/PD‐L1 blockade based on immune microenvironment status, 15 patients given immunotherapy including nine patients with *EGFR* Ex20ins and six with *HER2* Ex20ins were included in the present study. Among *EGFR* mutants, the ORR was 22.2% (2/9)，and one patient experienced stable disease with a PFS of 10.0 months. Both outcomes were confirmed by subsequent imaging. As for *HER2*‐mutant cases treated with PD‐1 inhibitor, the ORR was 0.0% (0/6). Notably, one patient experienced a disease hyperprogression (HPD) combined with a serious interstitial pneumonia, which was the cause of his death. The PFS for all individuals during immunotherapy and the representative CT images are shown in Fig 5.

## Discussion

To our knowledge, this study is the first to report the genomic and immune microenvironment features in NSCLC patients with Ex20ins of *EGFR* or *HER2*. In our dataset, *EGFR* Ex20ins alterations were identified in 35 cases (6.9% of *EGFR*‐mutant NSCLC and 2.8% of all NSCLC), and *HER2* mutations were detected in 1.7% of NSCLC patients. *EGFR* and *HER2* mutations were associated with younger population, female sex, and never‐smoker status, which was similar to previous observations in patients with sensitizing *EGFR* mutations.^21^ Overall, patients with *EGFR* or *HER2* Ex20ins had similar clinical characteristics to those with common *EGFR* mutations.

CGP identified A767_V769dup variant was the most common mutant type in lung tumors with *EGFR* Ex20ins. Co‐occurring mutations were not uncommon including *TP53* (45.0%), *PIK3CA* (20.0%), *CDKN2A* (10.0%), and *EGFR* amplification (20.0%). In addition, A775_G776insYVMA variant was the most common type among *HER2* Ex20ins and had high frequency concurrent mutations including *TP53* and *PIK3CA*. In line with previous studies,^7^, ^8^, ^9^ the most common coexisting genomic alteration of Ex20ins was *TP53*, with the mutation of *PIK3CA* being considered actionable as a result of its association with the therapeutic efficacy of PI3K‐AKT‐mTOR pathway inhibitors.^22^ Notably, comutational landscape was analgous between *EGFR* Ex20ins and sensitizing *EGFR* mutation.

In light of the advent of immunotherapy for the treatment of NSCLC, there is an increasing knowledge about the limited efficacy of PD‐1/PD‐L1 inhibitors in patients with *EGFR*‐mutant NSCLC.^15^, ^23^, ^24^, ^25^ To date, PD‐L1 is the best biomarker to guide immunotherapy. Our study explored the distribution of PD‐L1 expression in patients with *EGFR* and *HER2* Ex20ins as well as the infiltration of TILs. PD‐L1 expression in patients with *EGFR* Ex20ins was significantly higher than for those with *HER2* mutations (48.6% vs. 19.0%, *P* = 0.027). Likewise, a previous study reported that exon 20 mutants had higher PD‐L1 expression (48%) than *HER2*‐mutant (23%) or sensitizing *EGFR‐*mutant (22%) lung cancers.^26^ Meanwhile, this study identified that *EGFR* exon 20 mutants had a higher PFS and ORR to immunotherapy than those with common *EGFR* and *HER2* mutations (PFS: 4.0 vs. 1.9 vs. 1.9 months; ORR: 19% vs. 0% vs. 9%; *P* < 0.05),^26^ which may ascribe to high PD‐L1 expression. Nevertheless, a recent article showed that 81.7% of lung adenocarcinoma patients with *EGFR* Ex20ins had positive PD‐L1 expression (>1%).^7^ The higher PD‐L1 expression might be influenced by diverse populations, PD‐L1 antibody assay, and limited tissue samples; however, results from larger studies are still warranted.

Since TILs density are considered to act as a prognostic parameter and a predictive marker for the efficacy of PD‐1/PD‐L1 inhibitors,^27^ we surveyed its relevance in NSCLC patients with Ex20ins. The distribution of CD4+ and CD8+ TILs was similar between *EGFR* and *HER2* mutant groups. However, neither the infiltration of CD4+ TILs nor that of CD8+ TILs showed prognostic value in our cohort, which was consistent with previous observations.^7^ Moreover, no relationship between PD‐L1 expression and TILs infiltration was found in patients with Ex20ins.

In addition to PD‐L1 expression and TILs infiltration, other features such as TMB also appear to associated with clinical benefit from PD‐1/PD‐L1 inhibitors.^28^ In this study, mean TMB of Ex20ins was 3.3 mutations/Mb. TMB measured by CGP is comparable to measurements by whole exome sequencing and typically higher in smoking associated lung cancer due to tobacco carcinogenesis.^8^ Jonathan *et al*. reported the TMB of patients with *EGFR* Ex20ins was relatively low by analyzing 263 cases (mean 4.3, range 0–40.3 mutations/Mb).^8^ Likewise, median TMB was lowest (2.6 mutation/Mb) in cases with del19/L858R, and highest (5.2 mutation/Mb) in cases with G719X.^29^ In addition, *EGFR*‐mutated lung cancer has been previously shown to have a lower TMB compared with *EGFR* wild‐type lung cancer.^30^ Collectively, TMB has also been reported to be low in *EGFR*‐mutant NSCLC cases, such as *EGFR* Ex20ins, probably reflecting non‐tobacco associated carcinogenesis.^8^

For *EGFR* and *HER2* exon 20 insertions, a rigid placement of the α‐C helix in the inward and the phosphate‐binding loop (P‐loop) into the drug‐binding pocket, caused steric hindrance of the drug‐binding pocket from two directions, increasing the difficulty of drug binding.^12^ NSCLC patients with Ex20ins rarely achieve clinical benefit from EGFR‐TKIs, with an ORR around 11% and PFS of 2.0 months.^31^ Due to limited efficacy, clinical trials focusing on lung cancers harboring Ex20ins are substantially necessary. A recent clinical trial studying TAK‐788 (*EGFR*/*HER2* exon 20 inhibitor) showed an ORR of 43%, disease control rate of 86%, and median PFS of 7.3 months.^14^ Another phase II trial of poziotinib in 44 patients with Ex20ins was evaluated, which showed an ORR of 43.0%, and median PFS of 5.5 months,^12^ but the updated ORR of 14.8% among 115 patients is dismal. In addition, a study from China evaluated pyrotinib in the treatment of *HER2*‐mutated NSCLC with an ORR of 53%.^11^ In the present study, one patient harboring a *HER2* Ex20ins received pyrotinib experienced a PR with PFS of 9.0 months. In addition, a number of new antibody‐conjugated drugs have also emerged. For example, T‐DM1 treatment in patients with *HER2‐*mutant lung cancer obtained an ORR of 44% and 5.0 months of median PFS.^32^ At present, the application of new compounds targeting *EGFR* and *HER2* exon 20 mutations is promising.

However, no EGFR‐TKIs are currently approved for these patients and their diversity of structures suggests that different insertion events may have divergent responsiveness to TKIs. Immune checkpoint inhibition with PD‐1 and PD‐L1 antibodies has revolutionized the treatment landscape of NSCLC.^27^ However, patients with *EGFR*‐mutant NSCLC may be insensitive to PD‐1/PD‐L1 blockade. A recent meta‐analysis showed no OS benefit compared to chemotherapy in this population.^15^ In our cohort, 15 cases received PD‐1/PD‐L1 inhibitors. Nine patients harboring a *EGFR* Ex20ins mutation showed two PR, whereas patients with *HER2* mutation showed limited response to immunotherapy. What is more, one patient experienced HPD together with serious interstitial pneumonia. In line with the efficacy showed previously,^26^ patients with *HER2* Ex20ins seemed to have a poor response to the PD‐1/PD‐L1 blockade when compared with those with *EGFR* Ex20ins. Notably, a clinical trial of PD‐1 inhibitor treating NSCLC patients with *EGFR* or *HER2* Ex20ins is being carried out in our center, and further research conclusions are expected.

Limitations to our work include its retrospective nature as it involved a limited number of patients. Thus, it is certain that prospective, preferably multiregion, studies must follow in order to draw definitive conclusions. Moreover, a CGP test was not applied to every individual due to sample accessibility. Finally, the IHC scores for PD‐L1 expression and CD4+/CD8+ TILs infiltration could be affected by specimen type and antibody assay.

In conclusion, our study confirmed that lung tumors harboring *EGFR* or *HER2* Ex20ins have clinical and genomic characteristics which resemble those carrying sensitizing *EGFR* oncogenes. Additionally, it is likely that patients with *EGFR* Ex20ins will benefit from exposure to immunotherapy, a finding based on PD‐L1 expression; while PD‐1/PD‐L1 inhibitors showed limited efficacy in *HER2* mutants, which might benefit from new therapeutic strategies such as poziotinib or pyrotinib. Taken together, to maximize this effort, it is important to consider the execution of multinational studies in clinical trials in order to assess efficacy, highlighting the possibility of new therapeutic strategies in this patient subset.

## Disclosure

None of the authors reported a conflict of interest related to the study.
